# “Progressing physically and mentally” – a qualitative investigation into perceptions of mental well-being and barriers to participation in a nine-week yoga program among older inmates in Germany

**DOI:** 10.1186/s40352-026-00445-w

**Published:** 2026-07-21

**Authors:** Martin Koddebusch, Bernd Gröben, Pamela Wicker, Milan Dransmann

**Affiliations:** Department of Sports Science, Bielefeld University, Bielefeld, Germany

**Keywords:** Prison, Correction, Health, Offender, Rehabilitation, Qualitative Content Analysis

## Abstract

**Background:**

This study examines how a structured yoga program impacts the mental well-being of older inmates in the German correctional system and identifies barriers to participation. Older individuals in custody represent a growing yet largely overlooked subgroup in the scientific discourse, despite being disproportionately affected by the psychological and physical strains of imprisonment. To explore how yoga may support coping resources and social connectedness in this context, eight inmates (M = 63 years) completed a nine-week program and participated in qualitative interviews.

**Results:**

Findings suggest that participants perceived meaningful improvements in mental well-being, including increased self-reflection, emotional relief, and enhanced self-efficacy. Many described reduced physical discomfort, better balance and mobility, and the integration of breathing and movement techniques into their daily routines. Socially, the program fostered connectedness by strengthening mutual support and more positive interactions within the group. Barriers to participation emerged at both intrinsic and extrinsic levels. Intrinsic barriers were primarily tied to age-related physical limitations. Extrinsic barriers resulted from institutional conditions, such as scheduling conflicts, limited availability of structured physical activity, and insufficient program continuity.

**Conclusions:**

Overall, the findings suggest that yoga can serve as a valuable resource for strengthening coping capacities and social connectedness among older inmates in Germany. At the same time, the results underscore that such benefits depend on careful, needs-oriented program staging that accommodates physical limitations and institutional constraints. Given that older inmates have received minimal attention in prior research, this study highlights both the rehabilitative potential of yoga in the German correctional context and the necessity of thoughtfully adapted implementation to ensure accessibility and meaningful impact.

**Supplementary Information:**

The online version contains supplementary material available at https://doi.org/10.1186/s40352-026-00445-w.

## Introduction

Imprisonment is associated with low psychophysical health standards among inmates (Gallant et al., 2015) with prisons being labelled as “generally sick places” (Battaglia et al., 2013, p. 5684). Therefore, individuals within prison population face higher morbidity and mortality rates in comparison to general population (Mannocci et al., 2015). This situation is ethically concerning and also problematic regarding the institutional primary goal of social rehabilitation (Müller, 2024), as mental health is recognized as a crucial factor in crime prevention and the promotion of prosocial behavior (Ghanbarzadeh & Mohamadi, 2012). Although health-related challenges in prisons have been broadly documented, considerably less is known about how interventions can be adapted to meet the needs of specific and particularly vulnerable subgroups, such as older inmates. This limited understanding of how to adequately address population-specific needs represents an important research gap and provides a central motivation for the present study.

Low health standards in prisons arise from inherent aspects of the correctional system, particularly the central deprivation of liberty: Inmates experience daily lives marked by monotony and a near-total loss of autonomy and control (Dransmann et al., 2021; Müller & Mutz, 2019). This contributes to a range of psychosocial and behavioral challenges, including social isolation (Müller & Mutz, 2019), unhealthy behaviors such as substance misuse (Battaglia et al., 2013), and an inactive lifestyle often accompanied by an irregular diet (Mannocci et al., 2017). These conditions are associated with anxiety, despair, social impairments, and mental illnesses such as depression (Ghanbarzadeh & Mohamadi, 2012), as well as physical health issues like obesity (Mannocci et al., 2015). Together, these dynamics – fundamentally characterized by the reduction of inmates to a childlike state of dependency – are conceptualized in the literature as the “Pains of Imprisonment” (Sykes, 1958/1974). These pains manifest as what is referred to as “prison sickness” (Koddebusch et al., 2025, p. 299), which in turn is marked by inner demotivation, paralysis, and listlessness, ultimately promoting inactive lifestyles and undermining both mental health and mental well-being.

For the purpose of this study, mental health is defined in line with the World Health Organization (WHO; 2026) as “[…] a state of mental well-being that enables people to cope with the stresses of life, realize their abilities, learn and work well, and contribute to their community”. The American Psychological Association (APA; 2026) expands this understanding by defining mental health as “a state of mind characterized by emotional well-being, good behavioral adjustment, relative freedom from anxiety and disabling symptoms, and a capacity to establish constructive relationships and cope with the ordinary demands and stresses of life”. These definitions indicate that mental health is not only the absence of mental illness, but also includes experiential, emotional, coping-related and social dimensions. Within this understanding, the term ‘mental well-being’ or ‘emotional well-being’ is positioned as a central component of mental health.

Well-being is commonly described in the literature as a broad and multidimensional construct (Zhang et al., 2024). In the context of sports within correctional settings, the term mental well-being is frequently invoked, yet often without a clearly articulated definition (e.g., Auty et al., 2015; Meek & Lewis, 2014). Drawing on the given definitions provided by the APA (2026) and the WHO (2026), Koddebusch et al. (2025, p. 2) proposed defining mental well-being as an essential element of mental health and precisely as “a quality of experience that is significantly shaped by psychological coping resources and social connectedness”. In line with this definition, these two components correspond to what may be conceptualized as personal experience quality – reflecting coping resources – and social experience quality – reflecting social connectedness. Thus, personal and social experience quality are used as analytical dimensions derived from the definition of mental well-being, rather than as separate clinical outcome measures. To ensure a consistent definitional framework, this interpretation of mental well-being serves as the conceptual basis for the subsequent analysis presented in this study.

The mental well-being of inmates is severely undermined by the conditions of imprisonment and is simultaneously considered an essential factor in promoting desistance from crime and supporting rehabilitation (Meek & Lewis, 2014). Therefore, correctional institutions must identify effective ways to enhance inmates’ mental well-being in order to meet rehabilitation goals. An essential tool in doing so is to offer inmates opportunities to meaningfully spend their leisure time, typically by integrating “[…] sports, cultural and artistic activities […]” (Basaran, 2016) into daily life. Stereotypical examples are a range of sports and exercise, games and other social activities, and television viewing (Truss, 2005). Among these, sports programs are considered to be the most popular option (Kuhn, 2019). Consequently, a promising approach is the integration of sports into daily prison routines, with sports referring broadly to structured forms of physical activity, including competitive and also non-competitive and health-oriented practices. As summarized in the systematic review by Woods et al. (2017), various sports programs have been shown to alleviate perceptions of the pains of imprisonment. Positive effects on physical, mental, and social health have been reported (Liguori & Calella, 2024; Bilderbeck et al., 2013; Herold et al., 2023), and improvements in mental well-being have likewise been associated with participation in sports (Koddebusch et al., 2025; Meek & Lewis, 2014; Ortega-Vila et al., 2020). Sports can also contribute to enhancing group cohesion and social interaction among inmates (Dransmann et al., 2024). In view of these benefits, sports are increasingly discussed as a potentially valuable means of achieving long-term rehabilitation outcomes (Kovalsky et al., 2020). However, empirical evidence directly supporting this assumption remains limited, as most studies focus on the incarceration period itself, while long-term research documenting post-incarceration periods, such as the study by Kovalsky et al. (2020), is still rare.

These positive outcomes cannot be assumed to emerge automatically from any sports program; rather, they depend on thoughtful structuring and careful adaptation to the specific target group (Müller & Schröder, 2023). Therefore, it should be noted that inmates may often be collectively referred to as the “prison population”, but this category encompasses several subgroups (e.g., male and female inmates, older and younger inmates) that differ substantially in their needs. Consequently, if programs are not didactically and pedagogically structured in an appropriate manner, unintended outcomes may occur (Müller & Schröder, 2023).

For example, Norman (2017) points out that sports can be instrumentalized both by inmates and by prison administration for their own purposes. Specifically, organized sports programs and recreational physical activities may provide inmates with a framework within which personal conflicts can be enacted under the guise of the respective activity, sometimes in violent ways. Physicality also plays a significant role in this dynamic: a muscular body achieved through intensive weight training functions primarily as a symbol of power, serving both as a means of self-protection and as a catalyst for the emergence of undesirable hierarchical structures.

Bahlo et al. (2022) address this issue as well, linking elevated levels of violence and the emphasis on muscular physiques to factors associated with toxic masculinity. In this sense, sport spaces may be reinterpreted by inmates as arenas for the development of hierarchical power structures and the violent negotiation of conflicts. These circumstances suggest that particularly competitive, strength- and physique-oriented activities associated with corresponding constructions of masculinity require deliberate reflection and intentional pedagogical framing within correctional settings as opposed to non-competitive, individual-oriented formats such as yoga and running. However, it remains important to acknowledge that such activities are not inherently problematic. Rather, it is primarily the existence of such subcultural orders and expectations that gives rise to problematic dynamics. Accordingly, a complete withdrawal from these forms of sport in correctional institutions would also not be a viable solution.

At the same time, Norman (2017) emphasizes that sport is also employed by prison administration as a mechanism of social control: “Sport and physical recreation are both a carrot and a stick in this regard, sometimes being viewed as an attractive activity for prisoners that can also promote good behaviour, and other times being withheld, as a form of punishment” (Norman, 2017, p. 603). Taken together, these considerations underline the importance of carefully differentiating between formats and their social dynamics when evaluating their potential within correctional settings. Building on these considerations, recent literature emphasizes that particularly vulnerable subgroups, such as older inmates, are often insufficiently reached by performance-oriented formats (Müller, 2024). Programs that prioritize competition may unintentionally reinforce exclusionary dynamics and counteract heterogeneous physical capacities. Accordingly, Müller (2024) and Müller and Schröder (2023) argue that sport interventions in correctional settings require explicit pedagogical framing, small-group organization, two to three sessions per week, and structured reflection phases to foster social learning and rehabilitative outcomes.

As stated previously, older inmates represent a vulnerable population, as their distinct physical and psychosocial needs are frequently overlooked. Given this, a needs-oriented and carefully staged program design is particularly important for the steadily growing subgroup of inmates over 50, who are disproportionately affected by the pains of imprisonment (Williams et al., 2012). Research indicates that incarcerated adults experience accelerated aging (Kaiksow et al., 2023), resulting in physiological aging that is estimated to occur 10–15 years earlier than in the general population (Joynt & Bishop, 2018). This further increases their vulnerability to health decline and underscores the relevance of targeted exercise interventions for this population. Wilkinson and Caulfield (2020) report in their systematic review that older inmates tend to exhibit an increased prevalence of physical and mental health conditions, including primarily cardiovascular, musculoskeletal, and respiratory diseases, mobility impairments, and depression. Evidence additionally shows that older inmates experience higher levels of comorbidity, mortality, chronic health conditions, and physical as well as cognitive impairments compared to younger prisoners (Skarupski et al., 2018; Williams et al., 2012). However, Wilkinson and Caulfield (2020) note that the literature should be cautiously interpreted, as the overall discourse focused on the needs of older incarcerated people is deficient. Although their proportion is increasing, older inmates still constitute only about 16% of the prison population in Western countries (e.g., Skarupski et al., 2018; Fleckinger & Schmidt-Semisch, 2025).

Sports-related research focusing on this subgroup is consequently scarce, resulting in a clear gap in knowledge regarding their population-specific needs. Accordingly, sports programs for this vulnerable subgroup should be designed to minimize barriers to participation. Barriers to participation in prison sports are known to exist both as intrinsic and extrinsic barriers, although corresponding literature remains limited (Brosens, 2013). Intrinsic barriers refer to personal factors such as motivation or individual circumstances. Brosens et al. (2017) found that many inmates preferred alternative activities over participating in physical activity programs, such as work assignments, receiving visits or taking walks. The phenomenon of “prison sickness” (Koddebusch et al., 2025, p. 299) may further reinforce such intrinsic barriers. Extrinsic barriers arise from institutional conditions, including structural, financial or resource-related constraints, as described by Meek and Lewis (2014). Brosens et al. (2017) specify this by reporting inmates not receiving an “[…] answer to their […] request to register […], and being on waiting lists […]” (Brosens et al., 2017, p. 187).

In light of these barriers, yoga appears to be a suitable option for older inmates, as it is not performance-oriented and follows a holistic approach aimed at “[…] harmonizing body, mind, and soul” (Schmitz, 2022, p. 17). Therefore, beyond its inclusion within the broader sports-science discourse, yoga is widely conceptualized as a mind-body practice not primarily focusing on competitive or physique-oriented aspects. Rooted in Indian philosophy, its central aim is to regulate mental activity and foster harmony between bodily and psychological processes (Feuerstein, 2008; Schmitz, 2022). Consistent with this perspective, the term ‘yoga’ is often translated to “union”, referring to integration at multiple levels: the intrapersonal level, involving the union of body, mind, and spirit; the extrapersonal level, encompassing the individual’s connection with others and the environment; and the spiritual level, involving engagement with questions of life, meaning and opportunities for personal growth (Schmitz, 2022, p. 6). Consequently, yoga seems to inherently oppose prison-specific stressors, as it equally incorporates physical activity, self-attention, and also social involvement.

The international discourse on yoga in correctional settings indicates that yoga may promote prosocial behavior and reduce stress and anxiety among inmates (Bilderbeck et al., 2013). Studies further suggest that it can alleviate symptoms of depression and enhance mental health (Harner et al., 2010) as well as psychological well-being (Auty et al., 2015). Griera (2017) reports rising popularity for yoga in penitentiary settings within various countries and notes its potential for “lowering depression and anxiety among prisoners by emphasizing its capacity to foster emotional self-control and self-esteem […]” (p. 96). Furthermore, Griera (2017) highlights the difference between other sports and yoga, emphasizing the potential of “transcendence experiences” (p. 96) through the spiritual aspect of yoga activities. Moreover, trauma-informed yoga has been shown to benefit especially vulnerable individuals, including prison population (Tibbits et al., 2021). Overall, yoga has been reported to compensate effectively for prison-specific stressors and may even contribute to reduced recidivism (Kovalsky et al., 2020). On a German national level, only one publication regarding yoga in the prison context exists: Schmitz (2022) reports mentally calming effects and a reduction of physical complaints among male inmates. The focus on older inmates is not to be found in publications within the international and national discourse.

Despite the promising indications outlined above, the effectiveness of yoga for older inmates cannot be assumed without empirical exploration. Developing interventions in correctional settings requires a reasoned understanding of population-specific needs and potential barriers to participation. Since mental well-being is shaped through personal and social experience quality, which in turn are characterized by psychological coping resources and social connectedness, it is essential to investigate these dimensions in detail. Accordingly, the present study addresses the following research questions (RQ):

- RQ1: Personal experience quality – How does the program affect psychological coping resources of older inmates?
- RQ2: Social experience quality – How does the program affect perceptions of social connectedness among older inmates?
- RQ3: Barriers to participation – What barriers to participation are being perceived by older inmates?

Based on the results regarding RQ1 and RQ2, reasoned statements about the effects of the program towards mental well-being among inmates may be derived. In order to address the RQs appropriately, a qualitative interview design was selected. Although various interpretations of well-being can be measured using quantitative questionnaires (e.g., Pouwer et al., 2000; Hills & Argyle, 2002), the construct of well-being may take on different meanings within the prison context. More specifically, the prison context is, as previously described, essentially characterized by pains of imprisonment, resulting in extraordinary mental distresses for inmates. Yoga may therefore be experienced as a special opportunity to counteract prison-specific stressors and ultimately take on meanings and functions that differ substantially from community-based programs. Quantitative measures could show whether participants report certain levels of well-being, but they cannot sufficiently capture how participants experience yoga under correctional circumstances, which aspects of the program they perceive as especially relevant, and how barriers to participation are perceived. In view of these specific circumstances and the scarce research on yoga among older inmates – particularly in Germany – it seems appropriate to begin by generating in-depth, experience-based insights.

## Methods

This study followed a concept-informed qualitative descriptive research design. A qualitative approach was chosen due to the exploratory nature of the research questions and the limited state of knowledge in this specific field. While sport-based interventions in correctional settings have gained increasing attention, no empirical research to date has focused on older inmates in German prisons. In addition, the conceptual discourse on mental well-being in correctional contexts remains conceptually fragmented and lacks population-specific clarity. Given this gap, the study aimed to generate in-depth insights into how older inmates perceive and experience a yoga-based intervention, particularly with regard to personal and social experience quality. The qualitative approach was considered most suitable to capture subjective perspectives, contextual dynamics, and barriers to participation. Ultimately, the study seeks to contribute to both conceptual clarification and practice-oriented recommendations for designing population-specific physical activity programs in correctional settings.

To ensure methodological care, the study followed the COREQ checklist (Tong et al., 2007), which comprises three core domains: research team and reflexivity, study design, and analysis and findings.

### Research team and reflexivity

The study was conducted as part of a cooperation between the Bielefeld-Senne penal institution and the Department of Sports Science at Bielefeld University. Two university-based researchers coordinated the collaboration, planned the intervention, and conducted the training sessions. Both are academically trained in sports-science and have professional experience in designing and delivering physical activity programs. In preparation for the intervention, they engaged extensively with yoga theory and practice by participating in and observing yoga sessions to ensure adequate familiarity with the specific approach implemented. They were supported by four student research assistants. The student assistants contributed to the planning of the sessions and, on a rotational basis (two assistants per session), primarily demonstrated the exercises. They were likewise prepared through engagement with yoga theory and practice and through practical experience. The researchers focused on providing verbal cues, monitoring participants’ performance, and adapting the exercises flexibly to individual needs. As the researchers’ background was in sport science rather than clinical or therapeutic practice, the intervention was focused on age-sensitive physical activity and mind-body practices, and explicitly not on a trauma-informed yoga approach. Correctional officers were not present during the training to maintain an unobstructed learning environment. To increase transparency, we note that a university-hosted large language model BIKI - Bielefelder KI Interface was employed only for linguistic editing and improving textual coherence in the preparation of the analysis report and manuscript. The model was not used in the development of session outlines, the transcription of audio-recorded interviews, the analysis of interviews, or any other practical or methodological components of the study. No parts involving methodological reasoning, theoretical interpretation, or data analysis were generated by the model.

### Study design

Originally, twelve male older inmates from an open prison branch were enrolled in the yoga program. Participants were informed about the intervention through an organized information session, posted notices within the facility, and direct personal communication. Participation in the yoga program was entirely voluntary. Participants were eligible if they were 50 years or older, and provided informed consent. No additional inclusion or exclusion criteria were applied. Open prison settings permit inmates to leave the facility for work or educational purposes and to return afterward, and good behavior may lead to gradual expansions of unsupervised release privileges (Höltkemeyer-Schwick & Seidler, 2020).

The intervention lasted nine weeks and consisted of two 60-minute sessions per week (Mondays and Thursdays), resulting in a total of 18 sessions. Each session took place within the correctional facility, in a spacious multipurpose hall used for various institutional activities. With originally up to 12 participants and four instructors present, the participant-to-instructor ratio was approximately 3:1. The intervention was based on a gentle, Hatha-oriented yoga approach selected for its structured yet adaptable format and its suitability for older adults with heterogeneous functional capacities. The curriculum was developed by the two main researchers. Across the nine-week intervention period, the main exercise component followed a progressive structure. While maintaining a consistent session framework, intensity was progressively increased through the introduction of more complex postures and longer holding times, while carefully avoiding overload. As intensity increased, exercises were continuously offered in differentiated levels of difficulty to accommodate varying physical abilities. To enhance accessibility, yoga mats and chairs were used during all sessions. Chair-based variations, reduced ranges of motion, and alternative posture options were provided. Participants were consistently encouraged to self-regulate intensity and to modify exercises according to their individual comfort and capacity. Each session followed a consistent structure: an arrival phase with breathing exercises to enhance mindfulness, a gentle mobilization segment, a main part focusing on strength and balance, and a concluding phase involving either muscle relaxation or guided imagery, followed by a brief collective reflection. A detailed breakdown of the session structure is provided in Table 1.

**Table 1:** Typical session structure

Phase (Duration)	Content / Examples	Target
Arrival & Breath Focus (~ 5–10 min.)	• Gong to open the session • Breath awareness • Guided breathing techniques (e.g. 4-6-6) • Mindful seated posture	• Ritualized opening of the session • Promotion of mindfulness • Internal grounding • Body awareness
Gentle mobilization (~ 10 min.)	• Shoulder rolls • Neck rotations • Cat-Cow (seated or standing) • Smooth spine rotations • Foot activation	• Warming up: →Mobilizing joints →Activating muscles →Preparing the body for subsequent exercises
Stability & Balance (~ 25–30 min.)	• Sun Salutations • Warrior poses • Tree pose • Standing balance • Chair pose • Plank • Bridge	• Development of strength, stability, balance, and flexibility • Creating movement experience
Relaxation / Guided imagery (~ 5–10 min.)	• Progressive Muscle Relaxation • Autogenic training • Visualization exercises	• Reducing physical and mental tension • Promotion of inner calm • Mental recovery from stressors
Closing (~ 5 Min.)	• Gong to end the session • Final stretching • Brief collective reflection	• Reflecting the experience (Feedback) • Ritualized ending of the session

Due to transfers (*n* = 1), early release (*n* = 2), or exceeding the maximum permissible absence of four sessions (*n* = 1), four participants did not complete the program. Thus, eight older inmates, with a mean age of 63.5 years (± 3.5; range: 61–71 years), completed the full program and participated in the post-intervention interviews.

Four days after the final session, interviews were conducted with all eight participants. Each interview was jointly conducted by two trained research assistants, who were equally involved in asking questions and facilitating the conversation. Interviewees were informed about the research topic, participated voluntarily in the interview study and provided their written consent. Participants were explicitly assured that their statements would remain confidential and would not be shared with correctional staff or facility administrators. Neither participants nor the facility administration expressed concerns regarding the audio recording. Because the interviewers and interviewees had become acquainted during the intervention, the interviews took place in a trusting atmosphere. All conversations were held in the visiting room with no other individuals being present, and were audio-recorded using a digital voice recorder (Olympus LS-14). Data were stored and processed in accordance with the data protection procedures approved by the ethics committee of [University]. To ensure consistency and comparability across all interviews, a semi-structured interview guide comprising open-ended questions developed by the two main researchers was used (see appendix). Drawing from the conceptual considerations, the interviews addressed mental well-being in the sense of personal experience quality and social experience quality as well as perceived barriers to participation. All interviews were transcribed manually by the first author in accordance with the transcription guidelines proposed by Kuckartz and Rädiker (2023). The transcription followed a smooth-verbatim approach, focusing on the semantic content of the spoken material while omitting paralinguistic details. To ensure accuracy, the transcripts were reviewed by the rest of the author team prior to analysis. All transcripts were anonymized before being imported into MAXQDA (Version 26.0.0) to conduct the content structuring qualitative content analysis.

### Analysis and findings

The interview data were analyzed using content-structuring qualitative content analysis following the approach outlined by Kuckartz and Rädiker (2022). This approach consists of several coding steps within a continuously iterative analytic process. Accordingly, the analysis process combined deductive and inductive procedures. Based on the conceptual framework and the research questions, a deductive category system was created comprising the following three categories:

1. Personal experience quality, comprising statements referring to coping resources and self-related perceptions in relation to prison-specific stressors.
2. Social experience quality, comprising statements concerning perceived connectedness, communication, and group dynamics
3. Perceived barriers to participation, comprising statements covering both intrinsic and extrinsic obstacles affecting engagement in the program.

The deductive categories served as the overarching analytical structure, as the collected data were initially coded deductively according to these predefined categories. After reviewing the entire dataset, the second step of the coding process comprised the development of inductive subcategories that were continually revised, merged, or expanded in iterative coding cycles until a stable category system emerged. The primary coding was conducted by the first author. To enhance analytic rigor and the credibility of qualitative analysis, selected transcripts and coding decisions were reviewed and discussed with the rest of the author team.

The final system consisted of the three deductive main categories that served an organizational function and did not contain coded segments themselves. Each main category comprised a set of inductively formed subcategories with directly coded segments. No new subcategories emerged in the last phase of coding, suggesting that thematic saturation had been achieved. The findings are presented along the structure of the three main categories.

To ensure transparency, all interview passages cited in the results section are labeled with an anonymized identifier consisting of an identification number (“ID”) and the corresponding position within the respective transcript (“pos.”).

## Results

**Fig. 1:**
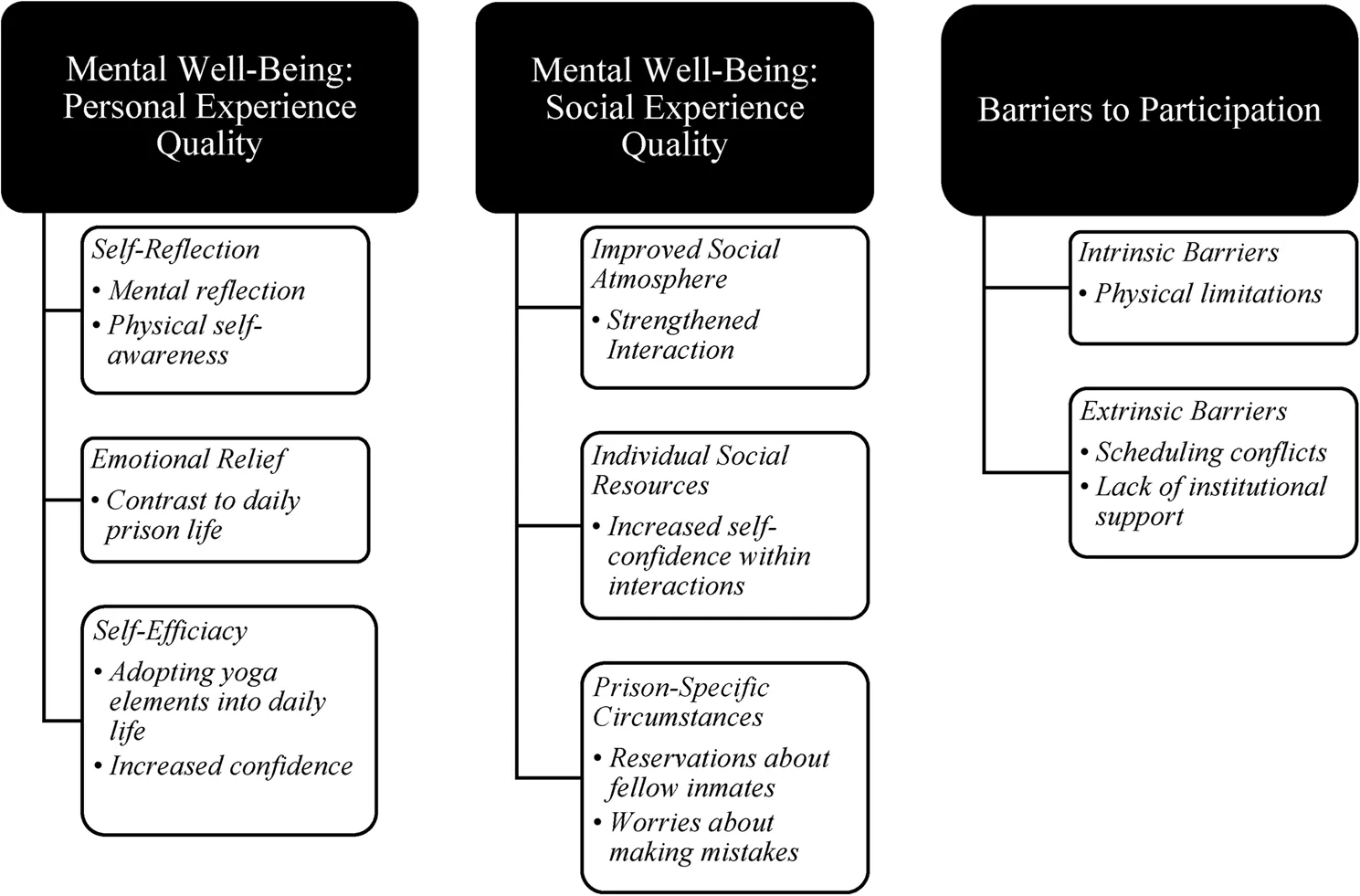
Main categories and subcategories of the analysis with key concepts. (Source: Authors’ own work)

The three deductively derived main categories were each differentiated into several inductively coded subcategories (Fig. 1). The applied concept of mental well-being is reflected in the first two main categories. The first category, personal experience quality, encompasses all statements referring to psychological coping resources in relation to prison-specific stressors (pains of imprisonment). The second category, social experience quality, includes statements concerning perceptions of social connectedness and the overall social climate within the facility. The third main category addresses perceived intrinsic and extrinsic barriers to participation in the yoga intervention. With an average duration of 38 min, the interviews provided a solid basis for generating in-depth insights.

### Mental well-being: personal experience quality

#### Self-reflection

The interviewees’ statements indicate that participants in the yoga program experienced an increase in self-reflection, both mentally and physically. Inmates frequently reported that the regular yoga sessions supported deeper contemplation of themselves and their circumstances: “You start thinking differently. […] You stop trying to avoid your worries and begin to face them — to face them in another way, so that you might be able to cope with them somehow” (ID 2, pos. 75). Similar experiences were described by others: “At least it showed me that it might be a way to deal with stress and worries — an additional way” (ID 9, pos. 142). Another participant particularly appreciated elements of the sessions that fostered such reflection: “It made me think. Maybe it’s because of this mental aspect of yoga, the relaxation parts that you don’t have in other sports. I liked that” (ID 1, pos. 182). He concluded that the program helped him “[…] to come to terms with myself again” (ID 1, pos. 38).

Regarding physical aspects, the yoga program introduced inmates to unfamiliar forms of movement and prompted them to reconsider their previous exercise routines and sporting histories: “Of course it’s very one-sided if you only ever cycle and do nothing else. It’s such a contrast, and it made me think: You’ve really missed out on a lot in terms of movement and sports” (ID 7, pos. 69). Since yoga was new to them, it posed a considerable motor challenge: “I realized that I had muscles in places I never thought about” (ID 1, pos. 85). Feeling their bodies so intensely was perceived as beneficial: “I realized that these movements had clear advantages. I physically felt better” (ID 8, pos. 8). This positive evaluation referred both to reductions in physical discomfort – “Basically […] the exercises […] made my complaints and difficulties fade or at least become weaker” (ID 7, pos. 110) – and to perceived improvements in motor performance, particularly balance: “[…] And the balance, too. It’s good for me, and I can really work on myself.” (ID 9, pos. 76).

#### Emotional relief

The interviewees described the yoga program as a welcome counterbalance to everyday life in prison (e.g., ID2, pos. 15; ID3, pos. 17; ID6, pos. 40; ID9, pos. 161). This counterbalance specifically manifested by achieving internal calmness that cannot be felt during typical prison days: “It helps by calming down. Turning off” (ID1, pos. 95). Other participants formulate it more specifically: “You could really concentrate in it. Really relax and just… There was no inner restlessness, absolutely not – at least not for the time of the session” (ID 7, pos. 38).

The need to achieve this kind of internal calmness stems from the interviewees’ perceptions of daily life in prison, which is marked by specific stress factors, primarily arising from deprivation of liberty and the loss of privacy: “[…] it’s […] difficult to find a moment here where you can be by yourself. Most people have at least one [inmate] in the room. […] There are hardly any places to retreat to” (ID1, pos. 21). With loss of privacy comes loss of autonomy: “They’re in charge, and you have to give in” (ID 3, pos. 94). Giving up self-regulation is described as “[…] the worst thing […]” (ID 9, pos. 53). The restrictions further influence possibilities to take responsibility for the respective social environment, as the interviewees describe: “Sometimes it scares me, when I feel relatively fine, but I know, my wife is not and that makes it difficult” (ID 8, pos. 34). Participants further described experiences of stigmatization by correctional officers, stating that they were frequently perceived simply as “[…] criminals – highly dangerous […]” (ID3, pos. 146), regardless of their actual individual circumstances.

Against this backdrop of daily strain, participants emphasized that the yoga sessions offered a rare mental space that allowed them to withdraw cognitively from the prison environment: “You come back to other thoughts and you are somewhere completely different” (ID3, pos. 31).

#### Self-efficacy

The interviewees reported that the yoga intervention fostered meaningful experiences of self-efficacy and increased self-confidence. A key topic was personal initiative: several inmates practiced yoga exercises independently outside the sessions, particularly balance and breathing techniques—“These are also exercises that I do in my room” (ID1, pos. 62). Breathing techniques became “automatic” in daily life (ID8, pos. 76). Personal initiative also appeared in sustained participation despite initial skepticism toward challenging exercises (ID3, pos. 129).

Participants emphasized perceptions of enhanced physical capability: “I can demand more from my body than I previously believed” (ID8, pos. 86). Exercises that initially seemed difficult were increasingly mastered: “I was amazed that I was able to do it so well” (ID9, pos. 65). Renewed confidence despite age was also highlighted: “[…] you don’t have to hide your mobility even at 61” (ID9, pos. 213). Persistence emerged as an important subject: “Just keep at it, try it, and don’t get discouraged” (ID7, pos. 59).

Participants described noticeable progress through yoga. Improvements in flexibility and balance were frequently mentioned: “You can move better” (ID6, pos. 77). Some reported health-related benefits, including reduced medication use: “From 150 milligrams of venlafaxine to zero – yoga helped with that” (ID1, pos. 26). Exercises initially perceived as impossible became achievable over time: “At the beginning I was a bit above the ground and later I could really put my hands on the ground […]. […] I was totally surprised that I would ever get there again” (ID7, pos. 51). Participants also described new perspectives – “a new attitude toward life” (ID7, pos. 44) – and practical relief in everyday activities, such as dressing or bending down (ID8, pos. 214). One participant summarized the overall experience as follows: “You notice that you are simply progressing physically and mentally” (ID7, pos. 77).

Inner activation and motivation were emphasized throughout. Participants reported overcoming their “inner weaker self” (ID1, pos. 45; ID8, pos. 137) and persevering despite challenges: “I didn’t always manage to persevere, but more and more often” (ID1, pos. 47). Yoga also motivated broader lifestyle changes, including increased physical activity and healthier eating (ID1, pos. 106). Participation was experienced as a commitment to follow through: “Because I started it once, I wanted to continue it to the end” (ID4, pos. 63). The perceived benefits further reinforced motivation: “I know every time how good the effect is, so I will try to keep using it for myself” (ID7, pos. 34).

### Mental well-being: social experience quality

#### Improved social atmosphere

Changes within the social atmosphere among the inmates were perceived by the participants. Those changes are based on the development of a fundamental community feeling: “You’re together with this big group and you don’t feel isolated at all.” (ID7, pos. 40). This togetherness fostered communication among inmates: “Suddenly you get together more often […]. You visit each other in the cells now. One could notice that” (ID9, pos. 192). Other participants reported similar experiences: “You said hello more and have also sat together more.” (ID3, pos. 127).

Another factor is the feeling of being inspired by other participants: “When you see the [inmate] […], I find it extraordinary that he was always there […]. But this is a 61-year-old man who is really physically handicapped, […] I find it fascinating where he gets the energy and the will to stand there and be there every time” (ID8, pos. 195). The interviewees report an increased willingness to help each other: “I also liked the team spirit, where one person helped the other when they couldn’t get up quickly, gave them a hand so that they could get up […]” (ID9, pos. 150). These impacts have been attributed to the non-competitive concept underlying the yoga program: “It was simply this shared experience […] without any sense of competition. It felt like a kind of team event in a way. The actual content isn’t even the most important part; it’s more about the concept and how you present it. Everyone made progress individually, but we also worked together as a group” (ID7, pos. 126). ID7 projects these positive outcomes on social atmosphere on one specific moment: “[…] we all carried the mats together and you could see how it brought the group closer together. It was like a kind of magic formula that kicked in” (ID7, pos. 129).

#### Individual social resources

Changes in social atmosphere also favored individual social resources of inmates: “I have become more self-confident towards the colleagues” (ID2, pos. 36). In this context, the interviewee also reports having become “[…] more sociable” (ID2, pos. 44). Another interviewee emphasized that the training group was perceived as a space that fostered positive emotions: “The people. That was always good for me […]. You are not alone. You have fun. The other one too. I had to laugh. All the time” (ID6, pos. 223).

#### Prison-specific circumstances

The interviewees repeatedly reflected on the social impact of the program against the background of prison-specific circumstances: “Originally, I had been closed off, […], you didn’t know these people. How do you approach them?” (ID 2, pos. 120). ID 7 emphasized that the group dynamic helped to “gain distance from personal conflicts” (pos. 36). Among the inmates. Another interviewee appreciated that the yoga instructors came from external institutions: “That was a psychological boost. Open and honestly. […] We get around a bit more with civilian people. That makes a big difference. When you have officers, they have their line. There’s no room for error” (ID3, pos. 123).

### Barriers to participation

#### Intrinsic barriers to participation

The interviewees’ perceptions of intrinsic barriers to participation were largely shaped by age-related physical limitations. One participant explained, “At first, I didn’t even want to, because I have terrible problems with my right shoulder. I’ve had osteoarthritis for three years and can hardly get my right arm over my head” (ID4, pos. 10). Comparable physical constraints were mentioned by other participants, including balance problems due to an inner-ear injury (ID9, pos. 67), nerve pain (ID8, pos. 27), chronic non-specific back pain (ID6, pos. 16), and evidence of prior surgical procedures (ID2, pos. 48). Another reflected more generally on the impact of aging: “When you get older, you sometimes think: Nothing works anymore—especially physically” (ID7, pos. 48).

#### Extrinsic barriers to participation

In terms of extrinsic barriers to participation, the inmates frequently discussed organizational constraints within the institution. As one participant explained, “[…] it was this schedule that fell right into this time, so for me, for example, I had to cancel my leave every time” (ID7, pos. 81). Similar scheduling conflicts were mentioned by others (e.g., ID8, pos. 70; ID6, pos. 189).

Beyond these organizational issues, the interviewees did not report additional extrinsic barriers – neither in relation to the yoga program nor to other potential activities – because no other sports programs were offered during their time in the facility. As one inmate stated, “Because we don’t have any programs here within the prison. There’s no one who takes care of anything” (ID7, pos. 133). Although the participants expressed interest in regular programs, several indicated that implementation was hindered by financial constraints on the institution’s side (e.g., ID8, pos. 185). One inmate recalled that an external occupational therapist had visited for a time, but the cooperation was discontinued “[…] because she was too expensive” (ID9, pos. 175). He concluded that “[…] when it comes to physical activity, people here are really just left on their own” (ID9, pos. 184).

## Discussion

The aim of the study was to examine how older inmates perceived the yoga program across dimensions of personal and social experience quality. By focusing on these experience qualities, the study provides insights into how yoga may impact psychological coping resources and social connectedness – two central dimensions of mental well-being. Perceived barriers to participation were also explored to better understand the institutional conditions shaping these experiences.

Regarding RQ1, the interviewees’ statements indicate that the yoga program strengthened self-reflection by fostering both mental reflection and physical self-awareness through exposure to unfamiliar forms of movement. Participants reported improved coping with worries (ID2, pos. 75), heightened bodily perception (ID1, pos. 85), reduced physical discomfort (ID8, pos. 8), and gains in balance and motor control (ID9, pos. 76).

The program also provided emotional relief by offering a clear contrast to daily prison life (ID2, pos. 15). According to the participants, daily routines in prison are shaped by prison-specific stressors, including primarily deprivation of liberty and loss of privacy (e.g., ID1, pos. 21), loss of autonomy (e.g., ID3, pos. 94), and restrictions in taking responsibility (ID8, pos. 34). Furthermore, participation in yoga encouraged inmates to adopt elements, such as breathing techniques and movement sequences, into their daily routines (e.g., ID1, pos. 62). This integration boosted their confidence in their physical presence and abilities, particularly in light of age-related concerns about declining skills (e.g., ID8, pos. 86). Yoga was therefore perceived as a way to overcome weak habits (ID1, pos. 45) and to support broader lifestyle changes, including increased physical activity and healthier nutrition (ID4, pos. 63). Therefore, the relaxation components described by participants may be understood as a structured counterbalance to prison-specific stressors.

Regarding accelerated aging and increased health decline (Kaiksow et al., 2023), such lifestyle changes appear desirable. These findings align well with existing literature, which positions yoga as a holistic practice integrating both personal and spiritual dimensions (Griera, 2017; Kovalsky et al., 2020). Schmitz (2022) highlights the harmonization of mind and body in yoga, an aspect also emphasized by participants in the present study (ID1, pos. 182).

Overall, the interviewees’ accounts suggest a reduction in psychological distress consistent with international findings (e.g., Auty et al., 2015; Harner et al., 2010). Kovalsky et al. (2020) point out that yoga programs may strengthen self-awareness and emotional regulation among inmates. While yoga-specific literature has previously linked yoga practice to increased self-esteem, self-improvement, and coping capacities in incarcerated and general populations (e.g., Griera, 2017; Kovalsky et al., 2020; Schmitz, 2022), the present findings extend this body of research by highlighting enhanced coping resources, self-efficacy, and self-confidence regarding physical abilities among older inmates.

The strong correspondence between the present findings and prior research suggests that inmates’ coping resources were strengthened through continuous participation in the yoga program. Drawing back to the underlying concept of mental well-being, essentially shaped by personal experience quality, the findings indicate that the yoga intervention fostered personal experience quality by enhancing coping resources.

In terms of social experience quality (RQ2), participants described improvements in the social atmosphere among inmates. These improvements were characterized by a strengthened sense of togetherness (ID9, pos. 192), increased communication (ID3, pos. 127), mutual support (ID9, pos. 150), respect (ID8, pos. 195), and shared non-competitive experiences (ID7, pos. 126). Participants also reported increased self-confidence in social interaction (ID2, pos. 36) and noted that the connectedness within the training group fostered positive emotions (ID6, pos. 223).

These findings align with the international discourse: Auty et al. (2015) emphasized in their systematic review that yoga may positively influence inmate behavior and prison culture. Kovalsky et al. (2020) highlight the potential of yoga in correctional settings to “acquire […] social strengths […] and social acceptance” (p. 14), indicating substantial potential for supporting social rehabilitation. Independent of the specific focus on yoga, sports programs in general are known to enhance social cohesion and social health among prison populations (Dransmann et al., 2024; Herold et al., 2023). The observation by ID7 (pos. 126) – that the development of social connectedness was not tied to the program’s content but rather to its non-competitive design – is reflected in the literature. In light of potential structural risks associated especially with competitive and strength- or physique-oriented formats discussed (Bahlo et al., 2022; Norman, 2017), the positive social developments observed in the present study appear linked to specific characteristics of yoga (Schmitz, 2022). Unlike competitive formats, which may under certain institutional conditions reinforce hierarchical dynamics or status-based comparison, the non-competitive and inward-oriented structure of yoga reduces performance pressure and minimizes opportunities for dominance displays. Furthermore, considering the specific characteristics of older inmates including accelerated aging (Joynt & Bishop, 2018; Kaiksow et al., 2023), increased morbidity, and heightened vulnerability to physical decline (Skarupski et al., 2018; Williams et al., 2012), adaptable and non-competitive formats may offer structural advantages. The emphasis on individual pacing allows participants with heterogeneous physical capacities to engage without reinforcing performance comparisons. In this sense, yoga does not merely function as another physical activity program but represents a format whose structural logic contrasts with both competitive sport cultures and prison-specific stressors. Müller and Schröder (2023) note, with respect to team sports in prisons, that the staging and didactic design of a program are more important than the specific content, and that rehabilitation-oriented reflection meetings are essential to foster desirable outcomes and avoid negative effects. Schmitz (2022) likewise points out that although research on yoga in the prison system remains limited, there is no evidence of negative or undesirable effects; instead, existing findings emphasize positive impacts that likely support social rehabilitation processes. She therefore concludes that yoga qualifies as a “low-threshold therapy offer” (p. 242).

In the present study, positive outcomes were emphasized particularly against the backdrop of prison-specific circumstances. These included initial interpersonal reservations arising from insecurities about fellow inmates due to their criminal status (ID2, pos. 120). The fact that the instructors came from an external institution was also appreciated, as it created a sense of civilian interaction and reduced the pressure inmates typically feel to avoid making mistakes (ID3, pos. 123). The idea of using sports programs as spaces to gain internal distance from the prison setting and create associative moments of liberty is reflected in the literature (Müller, 2024). Schmitz (2022) notes that the weekly yoga session was especially important to inmates, as it felt the least like the correctional institution compared to all other activities. Therefore, the findings indicate that social experience quality, in the sense of social connectedness among inmates, improved over the course of the yoga intervention.

Turning to RQ3, the interviews suggest that both intrinsic and extrinsic factors contribute to participation barriers for programs of this kind in correctional settings with older inmates. Regarding intrinsic factors, participants described a variety of age-related physical limitations (e.g., ID7, pos. 48). Since yoga programs involving older inmates have not yet been discussed in the literature, no empirical exploration of intrinsic barriers to participation in this specific context exists.

In terms of extrinsic factors, participants identified scheduling conflicts (ID6, pos. 189) and a lack of institutional support (ID9, pos. 184) as the primary barriers, emphasizing the absence of regular programs (ID7, pos. 133) and the facility’s limited financial resources (ID9, pos. 175). The deficient integration of sports programs into correctional institutions and the discontinuity of offerings due to financial constraints or low participation rates are frequently noted in the literature (Koddebusch et al., 2025; Meek & Lewis, 2014; Schmitz, 2022).

In summary, the study’s findings indicate that yoga may enhance both personal and social experience quality by strengthening inmates’ coping resources and perceptions of social connectedness. Based on the underlying concept of mental well-being, it appears reasonable to conclude that the program fostered mental well-being among older inmates. Nevertheless, given the range of age-related declines in physical abilities, programs should be designed to accommodate diverse performance levels and to provide opportunities for mental withdrawal from the correctional environment and its associated stressors. The interviews also suggest that such programs are not yet systematically integrated into institutional practice. In light of these findings and the empirical indications of rehabilitative potential, correctional facilities should critically examine their current practices.

## Limitations and practical implications

Even though the study revealed valuable insights, particularly regarding the needs-oriented design of yoga programs for older inmates and possibilities to strengthen their mental well-being, several limitations must be acknowledged. First, the study was conducted in a single correctional institution, which requires careful consideration. Correctional facilities can differ substantially depending on regional, structural, and organizational contexts. Consequently, the findings should be interpreted within the specific institutional setting. Nevertheless, the strong correspondence with international research on sports and yoga in correctional environments suggests that the results may be cautiously transferable to broader contexts (Schmitz, 2022).

A second limitation concerns the relatively small number of participants in both the yoga program and the interview sample, despite the achievement of data saturation. The findings should therefore be regarded as an exploratory first insight into a field that has received insufficient research attention to date. Regarding barriers to participation in particular, perspectives from non-participating inmates would have been especially valuable; however, interviews could only be conducted with individuals who voluntarily consented to participate. Access to other inmates was restricted due to institutional constraints and forced participation would not have been ethically acceptable. In this sense, the present study serves as a valuable starting point for examining a vulnerable subpopulation that has thus far been largely overlooked.

A potential third limitation relates to the dual role of the researchers as both yoga instructors and interviewers. This familiarity may have increased the likelihood of socially desirable responses. Participants might have emphasized positive aspects of the program or withheld criticism. This possibility should be considered when interpreting the findings. At the same time, the established acquaintance may have enabled more in-depth reflections than would have been possible in a more distant interviewer-participant relationship.

Considering these limitations, directions concerning future research can be deducted. First, future studies should examine yoga programs for older inmates across a broader range of correctional facilities and institutional contexts. Assessing both qualitative and quantitative measures within a broader field of research is needed to deepen the understanding of older inmates’ needs and to determine how these can be appropriately addressed in the design of yoga and other physical activity programs. Second, future research should explicitly assess general yoga-related outcomes, such as increased self-confidence, self-efficacy, and coping resources among older inmates, as these aspects emerged as relevant in the present study and have frequently been addressed in previous yoga-specific literature, but not yet sufficiently in relation to older prison populations. Also, female older inmates should be part of scientific research, as they barely receive scientific attention to date. Longitudinal or mixed-methods designs would be particularly valuable to examine the sustainability of perceived improvements. Third, research should explicitly include perspectives of non-participating inmates and additional stakeholders, including officers and administrators. Such perspectives may be especially relevant to the concept of barriers to participation. In light of persistent financial constraints in correctional systems, future research could also examine whether low-threshold or self-directed formats, such as yoga sessions delivered through instructional videos or printed materials, could represent a feasible supplement to guided group programs in fostering mental and physical health. However, such approaches cannot be assumed to be equivalent to guided interventions and would require careful empirical evaluation prior to broader implementation. In particular, questions concerning structural integration into institutional routines, professional supervision, quality assurance, and sustained participant engagement would need to be addressed within the specific organizational conditions of correctional settings.

Regarding practical implications, the most important step is the systematic integration of yoga programs into the daily routines of older inmates. Beyond the specific benefits of yoga, establishing regular access to any form of structured physical activity would represent a meaningful improvement. With respect to needs-oriented program design, interventions should account for the wide heterogeneity in performance levels and physical limitations while still providing opportunities for both physical and mental progress. In practice, this requires adaptable formats, such as chair-based and mat-based variations, and individual pacing and progression. Since yoga is based primarily on the union of body and mind, it is essential for programs in this context to be goal-oriented: the focus should not be on performance, but on mental relaxation in combination with health-oriented integration of physical activity into daily prison life. Therefore, instructors should be adequately trained to address the challenges associated with high levels of heterogeneity. First, instructors should have a clear understanding of the program goals so that these goals can guide the structure and delivery of the sessions. Second, instructors should be able to differentiate movements across several levels of difficulty to appropriately address the group’s differing capacities. In this sense, an understanding of movement progression and cueing is essential. Third, instructors should be qualified to work with older and potentially vulnerable participants, including the ability to safely adapt postures, to recognize pain or overload, and respond sensitively to age- and prison-specific conditions. Continuous communication between instructors and participants is essential and may be facilitated through regular feedback sessions or written evaluations. Such feedback can help instructors to monitor the balance between participants’ capacities and the program’s requirements, for example with regard to discomfort, individual difficulties with specific movements, and perceived strain. Furthermore, instructors gain insight into participants’ subjective perceptions during the sessions, which is essential for achieving program objectives. Reflective discussions of this kind may therefore enable instructors to continuously adapt the program structure, where necessary. They may also be important for participants, as they might help them to consolidate their experiences and increase the likelihood of transferring positive associations and elements of the program, such as breathing techniques, relaxation strategies, or physical activity routines, to other domains of life. Additionally, programs should be conceived as cooperative or at least non-competitive in nature. Creating a space that allows for associative moments of liberty is likewise important. This can be supported by minimizing typical correctional cues – for example, by involving instructors from external institutions or, when this is not feasible, by having officers wear plain clothing to foster a more civilian atmosphere.

Beyond these practical considerations, the findings also hold relevance for correctional policy. As the proportion of older inmates continues to grow, correctional systems will increasingly need age-appropriate health promotion strategies. Integrating low-threshold programs such as yoga into institutional routine should therefore be regarded not merely as an optional activity, but as a strategic component of rehabilitation and health care provision. Sustainable implementation requires long-term institutional commitment, stable funding structures, and reliable cooperation with internal or external providers. By prioritizing such programs, correctional authorities may support rehabilitation goals, reduce health-related burdens, and better address the needs of this vulnerable subgroup.

## Supplementary Information

Supplementary Material 1.

## Data Availability

The interview data are not publicly available due to confidentiality requirements and institutional restrictions. Anonymized excerpts can be provided upon reasonable request.
