# Supplementary material for: “Progressing physically and mentally” – a qualitative investigation into perceptions of mental well-being and barriers to participation in a nine-week yoga program among older inmates in Germany
Source: Health Justice. 2026 Jul 21;14:33. doi: 10.1186/s40352-026-00445-w (PMC13390324; doi:10.1186/s40352-026-00445-w)
Supplement: Supplementary file 1 — Supplementary Material 1. [file 40352_2026_445_MOESM1_ESM.docx]

Thank you for taking the time to participate in this interview. The questions will focus on mental well-being and will address, among other aspects, your personal and social experience quality, motivational aspects and barriers to participation. The interview will take approximately 30-45 minutes. To ensure that we do not miss anything, we will audio-record this interview. Everything we discuss will be treated confidentially and will only be used in anonymized form in our academic work. If you agree, we can begin.

**Introductory Questions:**

1. How are you feeling at the moment?
2. Before your incarceration, what was your general attitude toward yoga or relaxation exercises?
3. Prior to or during your incarceration, have you had any experience with yoga or relaxation exercises?
4. What were your initial thoughts when you heard that a yoga program would be offered in the correctional facility?
5. What were your reasons for participating?

**Personal / Social Experience Quality: Incarceration & Mental Well-Being**

1. How do you personally experience everyday life in prison?
   1. What characterizes life in this correctional facility?
2. Were there moments when yoga was particularly valuable for you in daily prison life?
   1. If so, in what way?
   2. If applicable: Did yoga influence your perception of time, isolation, or inner restlessness?
   3. If applicable: Do you feel that yoga influenced your sense of autonomy or control?
   4. Did yoga affect your self-confidence?
      1. If so, can you describe a specific situation in which you noticed this?
   5. If not, are there reasons for it not being valuable to you?
3. Was there a situation in which you discovered a new ability in yourself through yoga?
4. Are there things you feel more capable of today than before the yoga course, and have you noticed any changes in yourself?
5. How do you see yourself today compared to a few months ago?
   1. Physically and mentally?
6. When you think about your overall well-being, what does “feeling well” mean to you personally?
7. How have you generally felt during the past few weeks in prison?
8. How did you feel physically while incarcerated and during recreation time?
9. Were there specific exercises or moments in yoga that were particularly beneficial for you?
   1. For example, relaxation exercises, breathing techniques, or physical movements?
10. In what ways, if any, did yoga influence how you deal with stress or worries?
11. Did the yoga intervention influence your relationships with other participants or inmates?
12. How did you experience the group atmosphere during the yoga sessions?
13. Did participating in the program affect how you interacted with other participants?

**Barriers to Participation:**

1. What motivated you to participate in yoga on a regular basis?
2. Has your motivation in other domains - such as daily life, work assignments, or leisure activities - changed since practicing yoga?
3. What does yoga offer you that other sports or recreational activities do not?
4. Was there a specific obstacle you had to overcome in order to participate in yoga regularly?
5. Were there moments when you considered discontinuing your participation?
6. What could we have done to help you feel more comfortable or motivated?

**Activities in Prison Life and Future Perspectives:**

1. During your incarceration, have there been other programs or initiatives offered?
   1. If so, which ones? Were they attended?
   2. If not, why are they no longer offered?
2. Did the program influence your attitude toward physical activity, exercise, or sports programs?
3. Can you imagine continuing yoga or engaging in other sports after your release?
4. Are there sports or forms of physical activity you would like to try if you had the opportunity?
5. Do you think yoga provides you with something meaningful for life after incarceration?
   1. If so, what exactly?

**Final Reflection:**

1. If you briefly close your eyes and think about your time in the yoga program, which two key experiences come to mind first?
2. What would you say to someone who is unsure whether yoga is suitable for them?
   1. And what would you specifically say to other older incarcerated individuals?
3. What specific changes would you suggest for future yoga sessions to make them even more accessible and beneficial for everyone?
4. What types of sports or physical activity programs would you like to see offered in this facility in the future?
5. Is there anything else you would like to add?
